# Topical Solution for Retinal Delivery: Bevacizumab and Ranibizumab Eye Drops in Anti-Aggregation Formula (AAF) in Rabbits

**DOI:** 10.1007/s11095-024-03721-2

**Published:** 2024-06-05

**Authors:** Steven A. Giannos, Edward R. Kraft, Jonathan D. Luisi, Mary E. Schmitz-Brown, Valentina Reffatto, Kevin H. Merkley, Praveena K. Gupta

**Affiliations:** Department of Ophthalmology and Visual Sciences, University of Texas Medical Branch, Galveston, TX USA

**Keywords:** antibody, anti-VEGF, macular degeneration, neovascularization, retina

## Abstract

**Purpose:**

Wet age-related macular degeneration (AMD) is a blinding retinal disease. Monthly intravitreal anti-VEGF antibody injections of bevacizumab (off-label) and ranibizumab (FDA approved) are the standard of care. Antibody aggregation may interfere with ocular absorption/distribution. This study assessed topical delivery of dilute antibodies to the posterior segment of rabbit eyes using a novel anti-aggregation formula (AAF).

**Methods:**

Bevacizumab, or biosimilar ranibizumab was diluted to 5 mg/ml in AAF. All rabbits were dosed twice daily. Substudy 1 rabbits (bevacizumab, 100 µl eye drops): Group 1 (bevacizumab/AAF, n = 6); Group 2 (bevacizumab/PBS, n = 7) and Vehicle control (AAF, n = 1). Substudy 2 rabbits (ranibizumab biosimilar/AAF, 50 µl eye drops): (ranibizumab biosimilar/AAF, n = 8). At 14.5 days, serum was drawn from rabbits. Aqueous, vitreous and retina samples were recovered from eyes and placed into AAF aliquots. Tissue analyzed using AAF as diluent.

**Results:**

Bevacizumab in AAF permeated/accumulated in rabbit aqueous, vitreous and retina 10 times more, than when diluted in PBS. AAF/0.1% hyaluronic acid eye drops, dosed twice daily, provided mean tissue concentrations (ng/g) in retina (29.50), aqueous (12.34), vitreous (3.46), and serum (0.28 ng/ml). Additionally, the highest concentration (ng/g) of ranibizumab biosimilar was present in the retina (18.0), followed by aqueous (7.82) and vitreous (1.47). Serum concentration was negligible (< 0.04 ng/ml). No irritation was observed throughout the studies.

**Conclusions:**

Bevacizumab and ranibizumab, in an AAF diluent eye drop, can be delivered to the retina, by the twice daily dosing of a low concentration mAb formulation. This may prove to be an adjunct to intravitreal injections.

**Supplementary Information:**

The online version contains supplementary material available at 10.1007/s11095-024-03721-2.

## Introduction

Age-related macular degeneration (AMD) is a progressive, degenerative disease of the macula that ranks third among the global causes of visual impairment [1]. Clinically, AMD encompasses the dry form with drusen deposits or the wet form with additional choroidal neovascularization. Neovascularization causes abnormal growth of the choroidal blood vessels, leading to vascular leaks and progressive vision loss [2]. Anti-vascular endothelial growth factor drugs (anti-VEGF) can slow the propagation of abnormal vascular growth [3]. Current drug treatment protocols have poor ADME properties, that is the absorption, distribution, metabolism, and excretion of the drugs. Specifically, the most efficacious delivery method is an intravitreal injection, as topical applications typically do not absorb into the eye. However, due to risks of complications and risks of the intravitreal injections, the dosing frequency is limited to once a month, and therefore is only a bolus dose instead of sustained delivery.

The standard of care for choroidal neovascularization; i.e. wet-AMD, is a monthly invasive intravitreal injection of an anti-VEGF monoclonal antibody drug over many months [4–6]. Intravitreal injections of an anti-VEGF monoclonal antibody (mAb), are used in wet-AMD and diabetic retinopathy (DR) to counter the overexpression of VEGF that promotes neovascularization [7]. Technically, clinical intravitreal injections are a bolus dose of mAb drug placed into the vitreous, which then diffuses throughout the posterior segment. The antibody then binds with excess VEGF and is cleared by choroidal circulation [8].

Ranibizumab (Lucentis^®^) is a humanized anti-VEGF antibody fragment; FDA approved for the treatment of: neovascular (wet) age-related macular degeneration, macular edema following retinal vein occlusion, diabetic macular edema, diabetic retinopathy, and myopic choroidal neovascularization. Bevacizumab (Avastin^®^) is a recombinant humanized monoclonal IgG1 antibody, used off-label for proliferative (neovascular) eye diseases and diabetic retinopathy. Intravitreal pharmacokinetic studies, using 1.25 mg bevacizumab and 0.5 mg ranibizumab, showed mean vitreal resident time to clear (> 69%) of 5.92 days for bevacizumab and 4.03 days for ranibizumab [9]. Treatment courses are often repeated over several years to address the pathology that progressively leads to blindness. These interventions, although effective, are associated with the risk of further vision diminishing complications such as retinal detachment or endophthalmitis [10].

Non-invasive or less invasive methods of drug delivery show promise, but also significant limitations [11]. Enhanced transscleral drug delivery has been accomplished through energy-based systems including iontophoresis [12–14] sonophoresis [15–18] and photokinetics [19–21]. Chemical and peptide based drug carrier permeation technologies are being explored [22]. Ocular dug delivery, using eye drops and passive diffusion transfer remains the single least invasive system for a wide array of applications. Historically, the absorption and distribution of topically applied drugs are low. Consequently, a new approach to improving absorption through tissue barriers and distribution to the retina is required.

Effective topical drug delivery across tissue barriers, such as sclera, is diminished due to drug molecular size, polarity and solubility, as well as intrinsic properties of the tissue environment and drug concentration. Ranibizumab, is a monoclonal antibody fragment (Fab) created from the whole antibody bevacizumab. Ranibizumab is calculated as 2/3rd the molecular size (hydrodynamic radius) and 1/3rd the molecular weight of bevacizumab [23–26].

The issue of antibody aggregation is one of the several obstacles for the development of topically applied antibody based biotherapeutics as eye drops. Any resulting aggregates, with increased molecular weight and size, may prevent tissue permeation and therefore be less bioavailable. The phenomenon of reduced available active monomer IgG mAbs upon dilution in saline, phosphate buffered saline (PBS) as well as the manufacturer’s package formulation was only recently discovered (see Electronic Supporting Information for a description of Avastin^®^) [27]. Additionally, antibody drug was not detected in initial sclera permeation tests (1 Hr. application time), when PBS was used as the donor/receiver medium [28].

This inability to record and measure appreciable diffusing drug was the genesis of our entire effort to develop a composition that provided stable monomeric forms of anti-VEGF drugs in low concentrations, as previously described, using an *in vitro* Franz diffusion cell model. Transscleral permeation studies, using human eye donor sclera as the model membrane, demonstrated mAbs, compounded in AAF, traversed eye tissue in appreciable quantities. It is believed that a synergistic effect occurs between the antibody molecules, arginine, trehalose, and tween 80 that helps facilitate permeation [27, 28].

For *in vivo* studies, rabbit models were chosen due to the similarity to human ocular physiology and pharmacokinetics, easy handling and eyes large enough for tissue sample weight analysis by ELISA, as opposed to mice or rats. Additionally, rabbits do not have protein cross reactivity in the ELISA analytical methods for anti-VEGF drugs. Rabbits do not have the same retinal vasculature as humans, with an optic streak instead of an optic nerve head and foveal pit. While non-human primates are another viable model, they are cost prohibitive for a proof-of-concept study. Minipigs are an emerging alternative model but the lack of baseline data to compare against.

After successful completion of *in vitro* studies, and to evaluate the *in vivo* topical delivery of ranibizumab and bevacizumab to the posterior segment of rabbit eyes, using novel AAF diluent, an *in vivo* proof of concept study was conducted. The scope of this study is to demonstrate the improved antibody drug delivery using AAF; specifically increasing the absorption and distribution of the drug. Following the proof-of-concept study, future studies can explore the safety and efficacy of the formulations.

## Materials and Methods

### Materials

All chemicals used to prepare AAF and PBS were USP-NF and/or pharmaceutical grade. L-Arginine, sodium phosphate dibasic anhydrous, sodium phosphate monobasic monohydrate, sodium chloride and polysorbate 80 (Tween^®^ 80, low peroxide) was purchased from Sigma-Aldrich, St Louis Mo; α,α-trehalose dehydrate was purchased from VWR (Radnor, Pa). Hyaluronic acid (sodium hyaluronate) (Eye Drops Grade, HA-EM3.0) was obtained from Stanford Chemicals, Irvine, CA. Avastin^®^ (bevacizumab) 25 mg/mL was obtained from Genentech, South San Francisco, CA. Biosimilar ranibizumab at 22.1 mg/ml, in a formula equivalent to the marketed Lucentis product, was purchased from IchorBio, Wantage, Oxfordshire, England. Bevacizumab ELISA kits (AVA-E-U51) and ranibizumab ELISA kits (LUC-E-U52) were purchased from United Immunoassay, Agawam, MA.

### Formula Composition

The AAF formulation for eye drops, ELISA and eye tissue collection media was composed of: 0.3% NaCl (51.03 mM), 7.5% trehalose (198.24 mM), 10 mM arginine, 10 mM phosphates (ratio adjusted to a pH of 7.4), and 0.04%Tween 80 as described in Giannos *et al*. [27].

### Topical Eye Drops

AAF and PBS were supplemented with 0.1% ophthalmic hyaluronic acid. These solutions were degassed, sterile filtered, aliquoted, and stored at 4 °C until the day of study administration. Avastin^®^ (bevacizumab pharmaceutical composition, 25 mg/mL) was added to both eye-drop compositions to make a final concentration of 5 mg/ml and held at 4 °C in 900 µl low binding microfuge tubes. The stock ranibizumab biosimilar (22.1 mg/ml) was diluted to 5 mg/ml using the novel anti-aggregation formula (AAF) with 0.1% ophthalmic hyaluronic acid, then aliquoted into 900 µl low binding microfuge tubes. Treatment aliquots were held at 4 °C until used.

### Animal Studies

All animals were handled in accordance with Association for Research in Vision and Ophthalmology Statement for the Use of Animals and were approved by the Institutional *Animal Care and Use Committee (IACUC) of University of Texas Medical Branch,* Galveston, Texas, and according to the *AAALAC* oversight. As rabbits are USDA species, the power analysis upon building the study showed that an N = 8 animals with each eye treated as a technical replicate would fully power a feasibility study. N = 8 animals, with two eyes per animal is 16 eyes, and as stated in the methods, half of the eyes were topically treated, and the contralateral eye serves as the control. As is standard to avoid inter and intra subject variations, comparisons are first validated between treated and contralateral eye, then pooled into the respective treatment groups. For testing adverse effects or efficacy in a pathology model, the required N would be higher, and outside the scope of this study. Therefore, within the aims of this study, an N = 8 per group is sufficiently powered and conforms to *IACUC* and *AAALAC* guidelines.

### Substudy 1—Bevacizumab

**Fig. 1:**
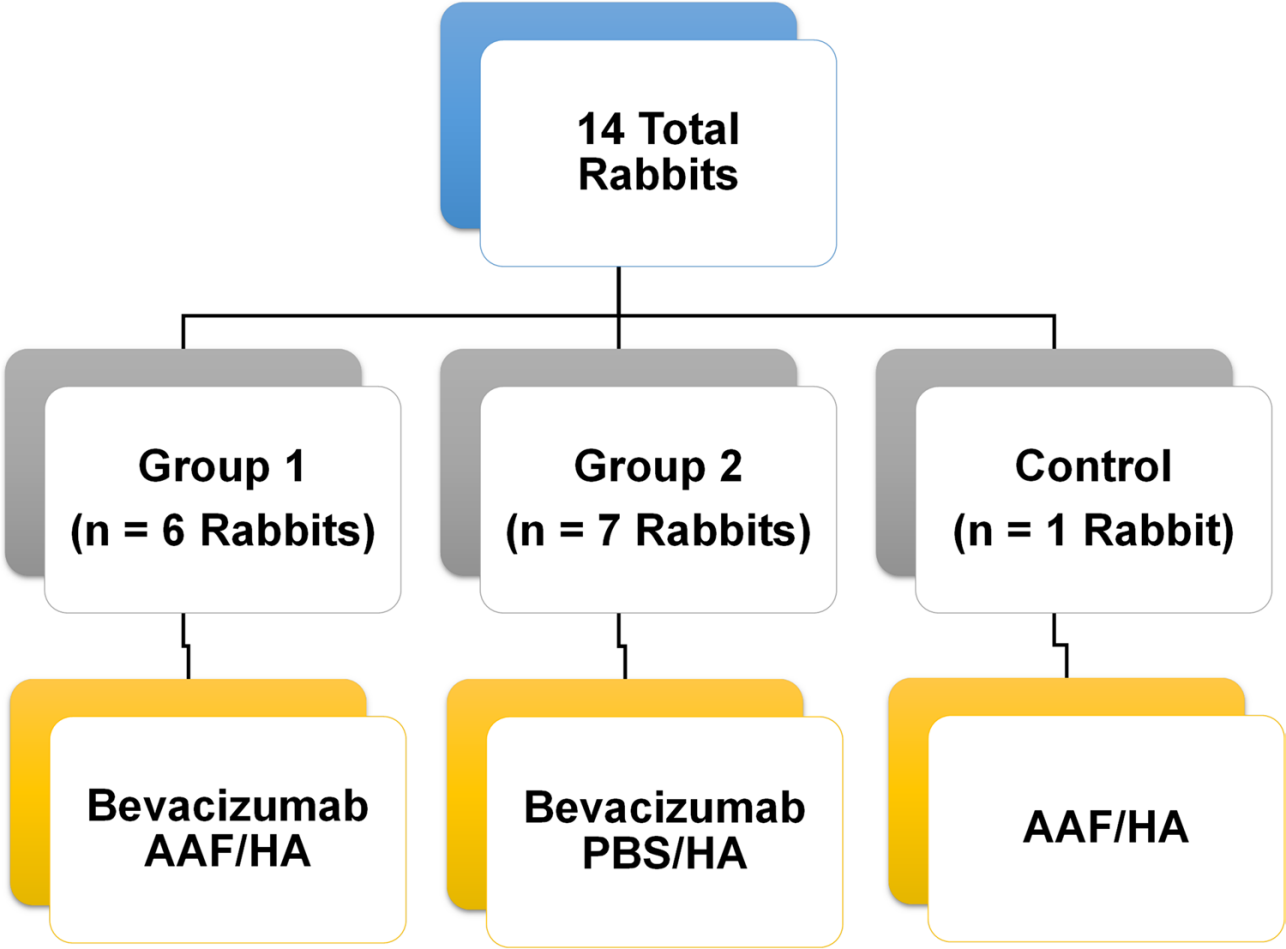
Schematic showing dosing groups for bevacizumab, substudy 1.

Fourteen New Zealand F1 rabbits (2–9 months old, no gender distinction) were housed under standard conditions; in a light-controlled room at 21 °C ± 3 °C and 54% ± 4% humidity. Food and water provided *ad libitum*. Animals were divided into 3 experimental groups, shown schematically in Fig. 1: Group 1—both eyes received a drop of topical (100 uL) bevacizumab in AAF/ 0.1% HA (5 mg/mL) twice daily (n = 6); Group 2—both eyes received a drop of topical (100 uL) bevacizumab in PBS/ 0.1% HA (5 mg/mL), twice daily (n = 7) and Group 3—both eyes received topical (100 uL) AAF/ 0.1% HA vehicle twice daily (n = 1). Animals were treated for 14.5 days. Dosing times were between 11–13 h between each dose.

### Substudy 2 – Ranibizumab Biosimilar

Eight New Zealand F4 rabbits (3–5 months old, 4 male & 4 female) were housed under the same conditions as substudy 1. Eye drops containing 50 µl of 5 mg/ml ranibizumab biosimilar in AAF were administered to both eyes of New Zealand rabbits (n = 8, age 3–5 months) twice daily. Animals were treated for 14.5 days. Dosing times were between 11–13 h between each dose.

At the end of both studies, 4 h after the last dose was given, the rabbits were placed under ketamine/xylazine anesthesia and blood was drawn for serum ranibizumab concentration. Rabbits were then humanely euthanized under deep anesthesia and eyes washed with PBS. Aqueous was removed by needle aspiration before enucleation. The eyes were then harvested and washed with PBS. Vitreous, and retina from each separate eye was recovered. The retina was peeled off the choroid. Choroid was not included in the study. Each tissue sample was placed into a previously prepared, individual tissue recovery aliquot of AAF to bring the final aliquot weight to about 300 mg; this being enough sample volume to plate samples in duplicate.

Individual tissue samples were analyzed by quantitative ELISA as per manufacturer’s instructions except for the substitution of the kit standard curve diluent with AAF and substitution of kit calibrator samples of bevacizumab or ranibizumab with study stock preparations as previously described [27]. Standard curve calibration dilutions were made to be in the lower range of the individual ELISA kit methods. Serial dilutions were prepared from the stock 25 mg/ml bevacizumab and from the stock 22.5 mg/ml ranibizumab biosimilar in AAF from 128 to 0.5 ng/ml and using unmodified AAF as the negative control.

Standard dilutions were used to establish the analytical curve relating optical density to concentration. The mathematical standard curve was applied to the optical density determinations of individual tissue samples and then multiplied by the dilution factor derived from the tissue weight and the weight of the AAF in the tissue recovery aliquot amount. See Supplementary Information for manufacturer’s mAb formulation, tissue preparation and ELISA protocol.

### Statistical Analysis

As appropriate the samples were quantified and sorted in Excel (Microsoft), and statistics and plotting in OriginPro^®^ (OriginLabs, Northampton, MA). As appropriate, the student’s t-test was used in simple comparisons and a 1-way ANOVA used in multiple condition comparisons. In all tests, statistical significance was determined with a p-value of < 0.05. With the ANOVA power analysis also being performed to ensure the statistics has a reasonable ‘n’.

## Results

### Substudy 1—Bevacizumab

**Fig. 2:**
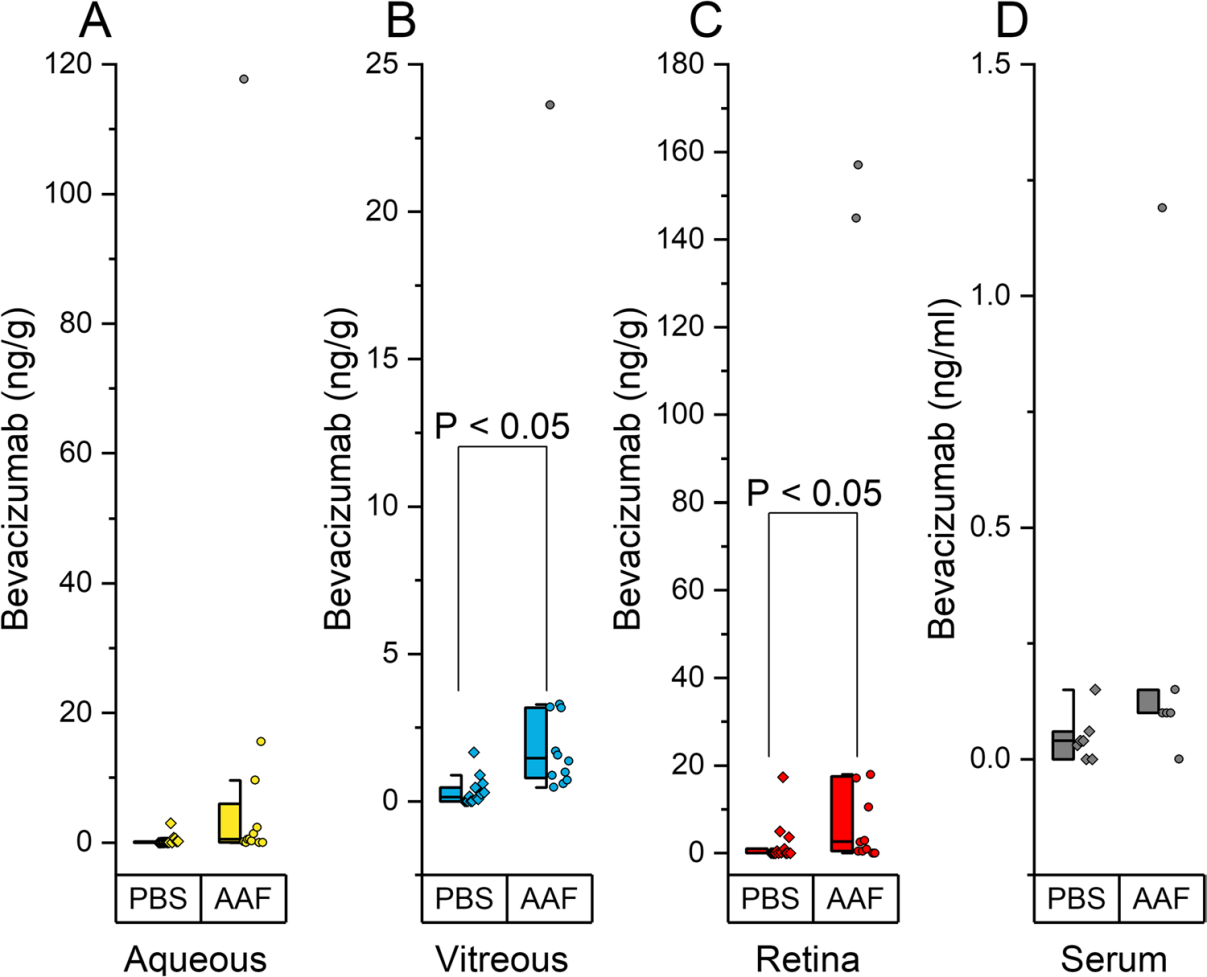
ELISA quantification of rabbit tissues after 14.5-day administration of 5 mg/mL bevacizumab in AAF (*n* = 6) compared to a PBS carrier (*n* = 7). **A** shows aqueous concentration, **B** shows vitreous concentration, 2c shows retina concentration and 2d shows serum concentration of bevacizumab. AAF facilitated significantly greater tissue concentrations compared to PBS.

In this study we compared AAF and PBS as the diluent vehicles for ocular delivery of bevacizumab. Hyaluronic acid was used, in both solutions, as a mucoadhesive to prolong eye drop contact time. Table I summarizes the dosing and findings of bevacizumab recovery in the rabbit aqueous humor, vitreous humor, retina, and serum. Group 1 that received the drug in AAF/0.1% hyaluronic acid, recovered mean tissue concentrations of bevacizumab in retina 29.50 ng/g *versus* 1.96 ng/g in PBS, aqueous humor 12.34 ng/g *versus* 0.34 ng/g in PBS, vitreous humor 3.46 ng/g *versus* 0.32 ng/g in PBS, and serum 0.28 ng/ml *versus* 0.04 ng/ml respectively. Figure 2 shows bevacizumab, when diluted in AAF, facilitated significantly greater tissue concentrations (10 times higher) in rabbit aqueous humor, vitreous humor and retina than when bevacizumab was formulated in PBS. No rabbit eye irritation or redness was observed throughout the study.

**Table I:**
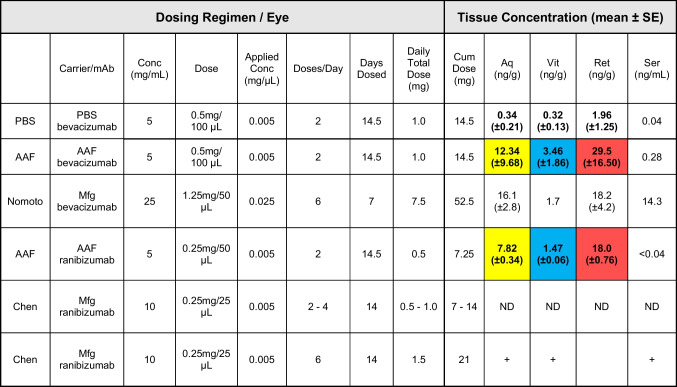
Results and *In Vivo* Effectiveness of Antibody Delivery Using AAF, as Compared to Previous Results Using the Manufacturer’s Formulation

(ND=None detected. + indicates trace detected but not quantitated). Nomoto *et al* [37], Chen *et al* [46]

### Substudy 2 – Ranibizumab Biosimilar

**Fig. 3:**
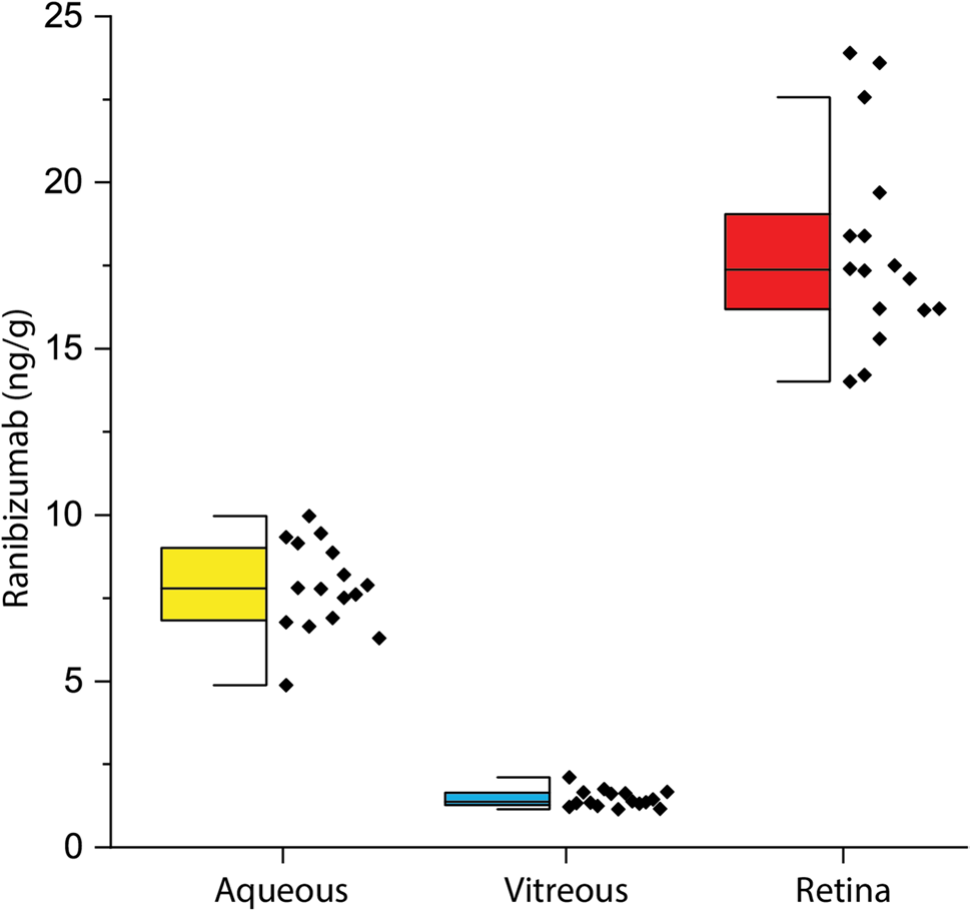
Comparison of the average ranibizumab biosimilar concentration as recovered in the each of the rabbit ocular tissues. ELISA quantification, *N* = 8 (16 eyes). Data represents tissue concentrations.

The dosing and findings of ranibizumab biosimilar recovery are shown in Table I. Ranibizumab was recovered in appreciable amounts in the treated rabbit eyes. Figure 3 summarizes the mean tissue concentrations (ng/g) of ranibizumab recovered from serum, aqueous humor, vitreous and retina. The highest concentration of ranibizumab biosimilar was recovered from retina (18.0 ng/g) followed by aqueous humor (7.82 ng/g) and vitreous (1.47 ng/g). Gross examination of the rabbit eyes showed no redness or discharge. Additionally, ranibizumab was negligibly present in the serum of the rabbits (< 0.04 ng/ml). A control of AAF without ranibizumab would be null.

### ANOVA Statistical Analysis

Statistical 1-way ANOVA analysis of substudy 1 and substudy 2, were performed using OriginPro®. For substudy 1, box plots of the concentrations of bevacizumab in the four compartments were constructed and shown in Fig. 2. The vitreous and retina concentrations, when compared, were significantly different; t-test *P* < 0.05. The AAF carrier did not have a normal distribution. The maximal values exceed the normal distribution. This may be due to variances in topical application, molecular weight, hydrodynamic volume, and drug resident time on the tissue surfaces. For ranibizumab, shown in Fig. 3, the treated groups are significant with *P* < 0.05, *N* = 16 eyes per group and the study is fully powered.

## Discussion

This study demonstrates the *in vivo* application of two major anti VEGF drugs to the posterior segment of rabbit eyes using twice a day dosing when diluted in AAF formula. The significant recovery of bevacizumab and ranibizumab in the rabbit eyes acts a proof of concept for topical mAb drug delivery that may change the paradigm of wet AMD treatment. Specifically, we demonstrate that the absorption and distribution of antibodies to the retina can be improved by twice daily dosing and by using a low drug concentration in AAF/ HA mucoadhesive carrier.

Systemic administration of drugs for posterior segment diseases is inefficient, with poor bioavailability within the immune privileged eye, due to blood-ocular barriers of tight junctions that normally protect the eye from circulating antigens, inflammatory mediators, and pathogens. The barrier has evolved to confer protection to the delicate microenvironment of the retina, and the tight junctions located between adjacent microvascular endothelial cells. The eye-blood barrier properties can restrict the passage of up to 98% low-molecular-weight drugs rendering systemic delivery for eye targets ineffective [29]. Therefore, systemic administration allows for higher absorption, but low specificity and presents an even lower distribution to the target organ, in this case the retina.

Theoretically, drug delivery to the posterior segment of the eye can be achieved by two distinct methods. The first, by injecting a bolus and/or controlled release delivery system to the vitreous humor and allowing the dug to diffuse to the retina, the target tissue. This type of therapy can be thought of as “inside to outside” delivery. Intravitreal injection of drugs is such an example and is the current standard of care. Though effective, intravitreal injections are painful, stressful and may have additional vision threatening side effects [30] such as retinal detachment, intravitreal hemorrhage and infection [31–33]. Non-invasive and minimally invasive extended-release implants have been pursued to deliver anti-VEGF antibodies to the ocular posterior segment, but have not advanced due tolerability issues [8, 34].

The second method of delivery is by topical or “eye drop” application—with topical absorption and diffusional distribution to the retina. This route is peri-ocular, as well as transscleral and can be thought of as “outside to inside” delivery. Topically applied anti-VEGF mAb eye drops have been screened in ocular studies; finding the approach difficult, but feasible [35–43]. Herein we present the outside to inside method with quite a significant delivery to the posterior segment of rabbit eyes.

Passive ocular permeation is the primary driving force in drug penetration and is concentration, molecular weight and tissue contact time dependent when used in an eye drop dosage form. Permeation decreases as molecular weight and hydrodynamic size increases. Therefore, eye drop compositions need to be modified and optimized in order to reduce protein aggregation, maximize tissue contact time, and preserve the bioactivity of the biopharmaceutical [44, 45].

Previously we reported that Avastin, Lucentis and Eylea, when diluted in PBS or the manufacturer's formulation, lose 40–50% of their monomer concentration and drug activity as compared to the antibody drugs diluted in AAF. AAF retains the antibody in an active monomeric form, protects from dilutional monomeric loss and corrects absolute quantification in *in vitro* diagnostics (IVD). Though, the mechanism of action is not yet known, it is suggested that arginine, trehalose and Tween 80 work together in a synergistic fashion to isolate and stabilize the biopharmaceutical proteins. The formulation also disaggregated mAb aggregation while preserving the full binding activity. In addition, degassing the composition maximizes antibody monomer concentrations [27].

Additionally, we have also shown that AAF facilitated permeation of the three anti-VEGF drugs (ranibizumab, aflibercept and bevacizumab) through human sclera in a Franz cell model and exhibited an inversely proportional molecular weight dependent flux [28]. These *in-vitro* results led to investigations of passive, topical delivery of bevacizumab and ranibizumab in naïve rabbit eyes.

In the current study, AAF and PBS were first compared as diluents, to assess that AAF facilitated *in vivo* ocular mAb permeation in rabbit eyes. AAF and PBS with 0.1% hyaluronic acid compositions contained 0.5 mg/100 μL bevacizumab/drop (5 mg/mL) and when dosed twice daily on rabbit eyes provided a total of 1 mg/day (see Supporting Information).

Bevacizumab, when diluted in AAF, accumulated in rabbit aqueous humor, vitreous humor and retina 10 times higher than when diluted in PBS and with negligible non-target serum levels, shown in Table I. Figure 2 shows the resulting recovered eye tissue concentrations of bevacizumab in the rabbit aqueous, vitreous, retina and serum.

An additional rabbit study was then conducted using ranibizumab biosimilar at a reduced concentration. Ranibizumab biosimilar AAF with 0.1% hyaluronic acid compositions contained 0.25 mg/50 μL ranibizumab biosimilar/drop (5 mg/mL) and when dosed twice daily on rabbit eyes, provided a total of 0.5 mg/day. Again, hyaluronic acid was used as a mucoadhesive to prolong eye drop contact time. Ranibizumab biosimilar was recovered in appreciable amounts in the treated rabbit eyes. Figure 3 summarizes the mean tissue concentrations of ranibizumab recovered from retina, vitreous, aqueous and serum. Here, the recovered amounts are approximately 50% lower. This was expected, due to the concentration of the ranibizumab biosimilar dosing solution being 50% of the concentration of the bevacizumab dosing solution. These results, showing 1) modest drug concentration in the aqueous, 2) much lesser drug amount in the vitreous, 3) 2.3 times the aqueous drug concentration in the retina, and 4) minimal drug in the serum, substantially supports the hypothesis of peri-ocular and transscleral delivery by topical eye drops.

A review of the literature revealed only one study, Nomoto *et al*. [37], in which bevacizumab was tested as a passive eye drop in rabbits [37]. An additional study in rabbits by Chen *et al*. [46], described the topical application of ranibizumab [46]. Both studies used the Genentech marketed strength and pH of bevacizumab and ranibizumab (25 mg/ml, pH 5.91 and 10 mg/ml pH 5.32 respectively) as dosing solutions, without any dilution, pH adjustment or mucoadhesive addition.

Bevacizumab, as received from the manufacturer, is formulated with about 158 mM of trehalose and 42 mM of phosphate with a final pH of 6.2 and a calculated osmolarity of about 210 mOsm/L. Additionally, ranibizumab biosimilar is received from the manufacturer with about 264 mM of trehalose and 10 mM of L-histidine providing a final pH of 5.5 and a calculated osmolarity of about 275 mOsm/L. The acidic pH is required to maintain shelf stability and to prevent mAb aggregation.

In the current study, both the AAF carrier composition and the PBS drug carrier were formulated at a non-irritating, normal eye pH of 7.4. Various reports of rabbit tear film osmolarity range from about 280.5 to 376.1 mOsm/L [47, 48]. Therefore, the osmolarity for the bevacizumab formula was adjusted to an osmolarity of about 321 mOsm/L. and the PBS drug carrier was adjusted to an osmolarity of about 291 mOsm/L.

Prior studies by Nomoto and Chen, applied the manufacturer’s formulation directly to the rabbit’s eyes. In these instances, the pH and formula osmolarity may have interfered with the potential for better antibody permeation. The manufacturer’s bevacizumab formulation had amounts of phosphate that may have prevented the tear film from adjusting the pH into a normal range. Also, histidine in the ranibizumab formula may have also prevented tear pH normalization. These acidic conditions may have led to increased tear production, drug washout and minimal tissue contact time with inhibited drug permeation.

Conversely, the AAF formulation stabilized the mAb in a normal pH range and eliminated low concentration antibody aggregation. Trehalose and hyaluronic acid eyedrops have been shown to stabilize tear film, which may have led to the overall improvement in tissue contact time and increased tissue deposition [49]. Additionally, degassing the solution eliminates dissolved gasses, microbubbles and oxygen which can initiate aggregation and/or excipient oxidation.

Nomoto *et al*. dosed bevacizumab 6 times per day for 7 days and Chen *et al*. dosed ranibizumab 6, 4, and 2 times per day for 28 days. Results from the present study are compared to the results from Nomoto and Chen and shown in Table I. The comparison between the studies details the aqueous, vitreous, retina and serum bevacizumab concentrations found in those tissues relative to the dosing regimens, eye drop concentration and daily total dose.

The two current studies using bevacizumab and ranibizumab biosimilar and several literature references, all using topical treatments are compared. As shown, Chen *et al*. recovered only trace amounts of antibody and Nomoto *et al*. recovered minimal values. Anti-aggregation formula (AAF) preserves the antibody’s functionality when applied topically to ocular tissues as well as delivering an overwhelming quantity to the retina, the target tissue.

While Nomoto *et al*. dosed a substantially higher concentration of bevacizumab (manufacturer’s preparation; 25 mg/mL) with 6 times a day dosing of 1.25 mg in a 50 μL drop, administering 7.5 mg/day total, the present study used AAF formula consisting of a concentration of 5 mg/mL with twice daily dosing of 0.5 mg/100 μL drop with a daily administered total dose of 1 mg/day. Chen *et al*. also dosed with the manufacturer’s concentration of ranibizumab (10 mg/ml) with 6, 4 and 2 times a day dosing, with only the 6 times a day dosing showing any accumulated drug in ocular tissues. Both earlier comparative studies dosed 6 times a day, which is a clinically cumbersome schedule.

Presently, AAF/ 0.1% HA at pH 7.4 provided higher achievable ocular tissue concentrations, even with fewer doses were given (twice daily being a more acceptable clinical dosing schedule) and with a less concentrated eye drop in a non-irritating formulation. The entirety of the applied topical formula, along with active ingredient stability and function, pH, osmolarity and diminished irritation potential may have a profound positive effect on overall pharmacokinetics in topical ocular drug delivery.

The strategy of anti-VEGF antibody therapy is to bind the over expressed VEGF within the eye [50]. VEGF is produced in the retina in response to inflammation or ischemia and stimulates neovascularization within the retina [2]. Several studies obtained human vitreous samples from wet-AMD and diabetic retinopathy patients and quantitated VEGF [51, 52]. Untreated neovascular AMD patients demonstrated a concentration of about 0.1 to 1.3 ng/mL (100 to 1,300 pg/mL) of VEGF in the vitreous. The present study indicates vitreal anti-VEGF antibody concentrations, from eye drops, comparable to the expressed VEGF found in patients. And, to the best of our knowledge, for retina, the target tissue, the expressed VEGF concentrations in AMD patients are unknown. Nevertheless, an excess of active anti-VEGF mAb (18 – 29 ng/g) can be delivered to the retina using AAF eye drops.

Based on the established studies, one bevacizumab mAb can bind with two molecules of VEGF and one ranibizumab can bind with one molecule of VEGF [7]. A great excess of anti-VEGF mAb is required for monthly intravitreal dosing in order to provide sustainable therapeutic activity, as the majority of the drug is cleared from the eye. Monthly intravitreal injections, over time, produce huge peaks and troughs of drug concentration within the vitreous, instead of a constant steady concentration. More frequent bimonthly injections would be more beneficial in raising visual acuity scores. However, patient compliance is problematic with this stressful regimen [53]. Ideally, a daily dosing of dilute anti-VEGF mAb drug would offer a continuous and constant VEGF binding potential; providing a therapeutic level with a much lower resident concentration of anti-VEGF mAb [54].

Ocular anti-VEGF efficacy within the eye was beyond the scope of the current drug delivery studies. Injury models with exogenously administered or over expressed VEGF from physical injury would bind the anti-VEGF antibody and convolute the attainable tissue permeation flux analysis for a first stage drug delivery study. We believe that the formulation allows for scleral penetration, as well as peri-ocular tissue, to act as a depot for the drug, allowing for accumulation and slow release over time. Possibly, in the future, labeled drug could be used to quantify the drug delivered to the retina and identify the paths of permeation.

In summary, once it was determined that PBS could not be used as a reagent with antibodies, a new diluent and course of action was developed, thereby facilitating antibody tissue permeation, measurement, and quantification. Step one was developing the AAF formula, with antibody, and it’s *in vitro* testing [27, 28]. Step two was to validate the formula’s protein stabilization qualities, *in vivo* with bevacizumab, and compare it against PBS. Step three was to additionally evaluate AAF, *in vivo* with ranibizumab, a smaller FDA approved drug.

## Conclusion

The present *in vivo* rabbit studies demonstrate a significant improvement in the passive delivery of the mAbs bevacizumab and ranibizumab to the posterior segment of the eye. Bevacizumab in AAF permeated and significantly accumulated in the aqueous humor, vitreous humor and retina; 10 times or more than when it was diluted in PBS. The AAF/0.1% hyaluronic acid composition with bevacizumab, or ranibizumab dosed twice a day (a clinically relevant dosing schedule) provided higher tissue concentrations in aqueous humor, vitreous humor and the retina than previously reported.

Future studies, directly addressing topical anti-VEGF application to retinal neovascularization and identifying drug delivery pathways should provide additional information concerning the safety and efficacy of the formulation and demonstrate a more constant drug delivery profile.

This non-invasive treatment could provide a more patient friendly adjunct that would enhance patient compliant therapy, supplement or extend effective anti-VEGF administration and reduce the number of monthly injections. Eye drops of anti-VEGF mAbs may offer better clinical efficacy compared to once-a-month bolus intravitreal injection alone.

### Supplementary Information

Below is the link to the electronic supplementary material.

Supplementary file1 (DOCX 19 KB)

## Data Availability

Data supporting the findings of this study are available in the paper and the supplementary documents.

## Abbreviations

AMD: Age related macular degeneration
ELISA: Enzyme-linked immunosorbent assay
mAb: Monoclonal antibody
PBS: Phosphate buffered saline
VEGF: Vascular endothelial growth factor

## References

[1] Pascolini D, Mariotti SP (2012). Global estimates of visual impairment: 2010. Brit J Ophthalmol.

[2] Ambati J, Atkinson JP, Gelfand BD (2013). Immunology of age-related macular degeneration. Nat Rev Immunol.

[3] Kovach JL, Schwartz SG, Flynn HW, Scott IU (2012). Anti-VEGF treatment strategies for wet AMD. J Ophthalmol.

[4] Stewart MW (2012). The expanding role of vascular endothelial growth factor inhibitors in ophthalmology. Mayo Clin Proc.

[5] Keane PA, Sadda SR. Development of anti-VEGF therapies for intraocular use: a guide for clinicians. J Ophthalmol. 2012. 10.1155/2012/483034. 10.1155/2012/483034 PMC3246783 22220269

[6] Weber M, Sennlaub F, Souied E, Cohen SY, Behar-Cohen F, Milano G, Tadayoni R (2014). Review and expert opinion in age related macular degeneration. Focus on the pathophysiology, angiogenesis and pharmacological and clinical data. J Fr Ophtalmol..

[7] Eandi CM, Alovisi C, De Sanctis U, Grignolo FM (2016). Treatment for neovascularage related macular degeneration: The state of the art. Eur J Pharmacol.

[8] Del Amo EM, Rimpela AK, Heikkinen E, Kari OK, Ramsay E, Lajunen T (2017). Pharmacokinetic aspects of retinal drug delivery. Prog Retin Eye Res.

[9] Bakri SJ, Snyder MR, Reid JM, Pulido JS, Ezzat MK, Singh RJ (2007). Pharmacokinetics of intravitreal ranibizumab (Lucentis). Ophthalmology.

[10] Falavarjani KG, Nguyen QD (2013). Adverse events and complications associated with intravitreal injection of anti-VEGF agents: a review of literature. Eye (Lond).

[11] Nayak K, Misra M (2018). A review on recent drug delivery systems for posterior segment of eye. Biomed Pharmacother.

[12] Eljarrat-Binstock E, Pe'er J, Domb AJ (2010). New techniques for drug delivery to the posterior eye segment. Pharm Res-Dordr.

[13] Pescina S, Ferrari G, Govoni P, Macaluso C, Padula C, Santi P, Nicoli S (2010). In-vitro permeation of bevacizumab through human sclera: effect of iontophoresis application. J Pharm Pharmacol.

[14] Chopra P, Hao JS, Li SK (2010). Iontophoretic transport of charged macromolecules across human sclera. Int J Pharmaceut.

[15] Razavi Mashoof A. High intensity focused ultrasound in ophthalmology : part one, transscleral drug delivery : part two, infrared thermography for scalable acoustic characterization, an application in the manufacture of a glaucoma treatment device. Human health and pathology. [Doctoral dissertation, Université Claude Bernard] Lyon I. English. ffNNT : 2014LYO10066ff. fftel-00996286f. 2014. https://theses.hal.science/tel-00996286/file/TH2014_Razavi-Mashoof_Arash.pdf.

[16] Shah R, Zderic V (2009). Ultrasound-enhanced drug delivery through sclera. J Acoustic Soc Am..

[17] Cheung ACY, Yu Y, Tay D, Wong HS, Ellis-Behnke R, Chau Y (2010). Ultrasound-enhanced intrascleral delivery of protein. Int J Pharmaceut.

[18] Suen WL, Wong HS, Yu Y, Lau LC, Lo AC, Chau Y (2013). Ultrasound-mediated transscleral delivery of macromolecules to the posterior segment of rabbit eye in vivo. Invest Ophthalmol Vis Sci.

[19] Kraft ER, et al. Photokinetic ocular drug delivery methods and apparatus. The Board of Regents, The University of Texas System, assignee. US Patent 8,948,863. 2015.

[20] Godley BF, Rowe-Rendleman CL, Kraft E, Kulp G (2013). Transsceral Drug Delivery to the Posterior Segment of the Eye.

[21] Godley BF, Kraft ER, Giannos SA, Zhao ZY, Haag AM, Wen JW (2015). Photokinetic drug delivery: Light-enhanced permeation in an in vitro eye model. J Ocul Pharmacol Th.

[22] Gote V, Sikder S, Sicotte J, Pal D (2019). Ocular drug delivery: Present innovations and future challenges. J Pharmacol Exp Ther..

[23] Li SK, Liddell MR, Wen H (2011). Effective electrophoretic mobilities and charges of anti-VEGF proteins determined by capillary zone electrophoresis. J Pharm Biomed Anal.

[24] Wen HE, Hao J, Li SK (2013). Characterization of human sclera barrier properties for transscleral delivery of bevacizumab and ranibizumab. J Pharm Sci.

[25] Hutton-Smith LA, Gaffney EA, Byrne HM, Maini PK, Schwab D, Mazer NA (2016). A mechanistic model of the intravitreal pharmacokinetics of large molecules and the pharmacodynamic suppression of ocular vascular endothelial growth factor levels by ranibizumab in patients with neovascular age-related macular degeneration. Mol Pharm.

[26] Hirvonen LM, Fruhwirth GO, Srikantha N, Barber MJ, Neffendorf JE, Suhling K, Jackson TL (2016). Hydrodynamic radii of ranibizumab, Aflibercept and bevacizumab measured by time-resolved phosphorescence anisotropy. Pharm Res.

[27] Giannos SA, Kraft ER, Zhao ZY, Merkley KH, Cai J (2018). Formulation stabilization and disaggregation of Bevacizumab, Ranibizumab and aflibercept in dilute solutions. Pharm Res.

[28] Giannos SA, Kraft ER, Zhao ZY, Merkley KH, Cai J (2018). Photokinetic drug delivery: Near infrared (NIR) induced permeation enhancement of Bevacizumab, Ranibizumab and aflibercept through human sclera. Pharm Res.

[29] Campbell M, Humphries MM, Humphries P (2013). Barrier modulation in drug delivery to the retina. Methods Mol Biol.

[30] Doguizi S, Sekeroglu MA, Inanc M, Anayol MA, Yilmazbas P (2018). Evaluation of pain during intravitreal aflibercept injections. Eur J Ophthalmol.

[31] Haddock LJ, Ramsey DJ, Young LH (2014). Complications of subspecialty ophthalmic care: endophthalmitis after intravitreal injections of anti-vascular endothelial growth factor medications. Semin Ophthalmol.

[32] Storey PP, Patel D, Garg S (2020). Endophthalmitis following intravitreal injection of anti-vascular endothelial growth factor agents. Can J Ophthalmol.

[33] Patel D, Patel SN, Chaudhary V, Garg SJ (2022). Complications of intravitreal injections: 2022. Curr Opin Ophthalmol.

[34] Moisseiev E, Loewenstein A (2017). Drug delivery to the posterior segment of the eye. Dev Ophthalmol.

[35] Williams KA, Brereton HM, Farrall A, Standfield SD, Taylor SD, Kirk LA, Coster DJ (2005). Topically applied antibody fragments penetrate into the back of the rabbit eye. Eye.

[36] Brereton HM, Taylor SD, Farrall A, Hocking D, Thiel MA, Tea M (2005). Influence of format on in vitro penetration of antibody fragments through porcine cornea. Brit J Ophthalmol.

[37] Nomoto H, Shiraga F, Kuno N, Kimura E, Fujii S, Shinomiya K (2009). Pharmacokinetics of bevacizumab after topical, subconjunctival, and intravitreal administration in rabbits. Invest Ophthalmol Vis Sci.

[38] Ottiger M, Thiel MA, Feige U, Lichtlen P, Urech DM (2009). Efficient intraocular penetration of topical anti-TNF-alpha single-chain antibody (ESBA105) to anterior and posterior segment without penetration enhancer. Invest Ophthalmol Vis Sci.

[39] Furrer E, Berdugo M, Stella C, Behar-Cohen F, Gurny R, Feige U (2009). Pharmacokinetics and posterior segment biodistribution of ESBA105, an anti-TNF-alpha single-chain antibody, upon topical administration to the rabbit eye. Invest Ophthalmol Vis Sci.

[40] Thiel MA, Wild A, Schmid MK, Job O, Bochmann F, Loukopoulos V (2013). Penetration of a topically administered anti-tumor necrosis factor alpha antibody fragment into the anterior chamber of the human eye. Ophthalmology.

[41] Moisseiev E, Waisbourd M, Ben-Artsi E, Levinger E, Barak A, Daniels T (2014). Pharmacokinetics of bevacizumab after topical and intravitreal administration in human eyes. Graef Arch Clin Exp.

[42] Wilkinson-Berka JL, Deliyanti D. The potential of anti-VEGF (Vasotide) by eye drops to treat proliferative retinopathies. Annals Trans Med. 2016;4(Suppl 1):S41. 10.21037/atm.2016.10.27. 10.21037/atm.2016.10.27 PMC5104608 27868009

[43] Gough G, Szapacs M, Shah T, Clements P, Struble C, Wilson R (2018). Ocular tissue distribution and pharmacokinetic study of a small 13kDa domain antibody after intravitreal, subconjuctival and eye drop administration in rabbits. Exp Eye Res.

[44] Kraft ER, et al. Antibody and protein therapeutic formulations and uses thereof. The Board of Regents, The University of Texas System, assignee. US Patent 11,692,027. 2023.

[45] Delplace V, Payne S, Shoichet M (2015). Delivery strategies for treatment of age-related ocular diseases: From a biological understanding to biomaterial solutions. J Control Release.

[46] Chen JJ, Ebmeier SE, Sutherland WM, Ghazi NG (2011). Potential penetration of topical ranibizumab (Lucentis) in the rabbit eye. Eye (Lond).

[47] Corsi F, Arteaga K, Corsi F, Masi M, Cattaneo A, Selleri P (2022). Clinical parameters obtained during tear film examination in domestic rabbits. BMC Vet Res.

[48] Lantyer-Araujo NL, Lacerda AJ, Mendonca MA, da Silva A, Dorea Neto FA, Portela RD, Oria AP (2020). Rabbit as an animal model for ocular surface disease, Tear osmolarity, Electrolyte, and tear ferning profiles. Optom Vis Sci.

[49] Sanchez-Gonzalez JM, Silva-Viguera C, Sanchez-Gonzalez MC, Capote-Puente R, De-Hita-Cantalejo C, Ballesteros-Sanchez A (2023). Tear film stabilization and symptom improvement in dry eye disease: The role of hyaluronic acid and trehalose eyedrops versus carmellose sodium. J Clin Med..

[50] Ferrara N (2004). Vascular endothelial growth factor: basic science and clinical progress. Endocr Rev.

[51] Malik TG, Khalil M, Gul R, Ahmad SS, Munawar S (2016). Serum versus vitreous VEGF A and central macular thickness in diabetic macular edema and the effect of intra-vitreal bevacizumab on these variables. Pakistan J Ophthalmol.

[52] Mesquita J, Castro-de-Sousa JP, Vaz-Pereira S, Neves A, Passarinha LA, Tomaz CT (2018). Evaluation of the growth factors VEGF-a and VEGF-B in the vitreous and serum of patients with macular and retinal vascular diseases. Growth Factors.

[53] Stewart MW, Rosenfeld PJ, Penha FM, Wang F, Yehoshua Z, Bueno-Lopez E, Lopez PF (2012). Pharmacokinetic rationale for dosing every 2 weeks versus 4 weeks with intravitreal ranibizumab, bevacizumab, and aflibercept (vascular endothelial growth factor Trap-eye). Retina.

[54] Agarwal A, Rhoades WR, Hanout M, Soliman MK, Sarwar S, Sadiq MA (2015). Management of neovascular age-related macular degeneration: current state-of-the-art care for optimizing visual outcomes and therapies in development. Clin Ophthalmol.

