# Supplementary material for: Topical Solution for Retinal Delivery: Bevacizumab and Ranibizumab Eye Drops in Anti-Aggregation Formula (AAF) in Rabbits
Source: Pharm Res. 2024 Jun 5;41(6):1247–56. doi: 10.1007/s11095-024-03721-2 (PMC11196329; doi:10.1007/s11095-024-03721-2)
Supplement: Supplementary file 1 — Supplementary file1 (DOCX 19 KB) [file 11095_2024_3721_MOESM1_ESM.docx]

**Supplementary Information**

**Novel Eye Drops for Retinal Delivery: Bevacizumab and Ranibizumab in Anti-Aggregation Formula (AAF) in Rabbits**

*Steven A. Giannos^1*^, Edward R. Kraft^1^, Jonathan D. Luisi^1^, Mary E. Schmitz-Brown^1^, Valentina Reffatto^1^, Elizabeth Urias^1^, Kevin H. Merkley^1^, Praveena K. Gupta^1*^*

^1^University of Texas Medical Branch, Department of Ophthalmology and Visual Sciences, Galveston, Texas

**1) Initial antibody formulation (as received from manufacturer).**

**Bevacizumab (Avastin^®^) (25 mg/ml)**

Genentech

Formulation: Each mL of solution contains 25 mg bevacizumab, α,α-trehalose dihydrate (60 mg), polysorbate 20 (0.4 mg), sodium phosphate dibasic, anhydrous (1.2 mg), sodium phosphate monobasic, monohydrate (5.8 mg), and Water for Injection, USP. The pH is 6.2. (Avastin® package insert) <https://www.gene.com/download/pdf/avastin_prescribing.pdf>

**Ranibizumab Biosimilar (22.1 mg/ml)**

**Research Grade [ICH4031]**

Ichorbio

Formulation: Sterile, colorless to pale yellow solution containing histidine hydrochloride, 10% trehalose dihydrate and Tween 20. pH 5.5. BSA and Azide free.

**2) Solution preparations**

The AAF formulation for ELISA analytical standards and for eye tissue sample collection media, described earlier (Giannos et al., 2018) contains 0.3% NaCl (51.03 mM), 7.5% trehalose (198.24 mM), 10 mM arginine, 10 mM phosphates (ratio adjusted to a pH of 7.4), 0.04% Tween 80 with the balance w/v being deionized water. The solution was vacuum degassed and sterile filtered with a 0.22 micron filtration unit. Solution was aliquoted and held at 4°C until day of use.

The base AAF formula was modified for eye drop use by the addition of 0.1% hyaluronic acid to the mixture and allowed to fully dissolve by stirring at room temperature. The solution was vacuum degassed and sterile filtered with a 0.22 micron filtration unit.

Under sterile conditions, bevacizumab pharmaceutical composition 25 mg/mL or ranibizumab 22.1mg/ml was added to the AAF eye drop composition to make a final mAb concentration of 5 mg/mL. Individual volume dosing aliquots of the AAF/mAb were made and held at 4°C until day of use.

A pharmaceutical grade PBS formulation was manufactured to mimic PBS used in prior experiments and adapted here for eye drop use comprising 137 mM sodium chloride, 2.7 mM potassium chloride and 11.9 mM phosphates ratio adjusted to a pH of 7.4, with the balance being deionized water. 0.1% hyaluronic acid was added to the mixture allowed to fully dissolve. The solution was vacuum degassed and sterile filtered with a 0.22 micron filtration unit. Under sterile conditions, bevacizumab pharmaceutical composition 25 mg/mL was added to the pharmaceutical grade PBS formulation eye drop composition to make a final mAb concentration of 5 mg/mL. Individual volume dosing aliquots of the PBS/ mAb were made and held at 4°C until day of use.

Tissue collection and ELISA diluent AAF formulation was comprised of 0.3% NaCl (51.03 mM), 7.5% trehalose (198.24 mM), 10 mM arginine, 0.04% Tween 80, 10 mM phosphate ratio adjusted to a pH of 7.4 with the balance being deionized water. The solution was vacuum degassed and sterile filtered with a 0.22 micron filtration unit. The solution was held at 4°C until day of use.

Aqueous, vitreous and retina AAF sample collection aliquots were prepared to so that each sample could be placed in the AAF composition with enough volume for ELISA quantification. Individual collection aliquots were prepared by weight for aqueous and vitreous 0.23 g (average) AAF and 0.27 g (average) AAF for retinal samples. After sample collection each aliquot was weighed to determine dilution multiplier coefficient for each sample. Average aqueous sample weight was 0.2416 g. Average vitreous sample weight was 0.8914 g. Average retina sample weight was 0.0761 g. Dilution multipliers for each sample were calculated and applied as a multiplier for each sample ELISA quantification result.

**3) AAF ELISA diluent and Validation of ELISA method**

ELISA bevacizumab and ranibizumab ELISA standard dilutions of mAb/AAF were prepared by diluting w/w the (same lot) bevacizumab pharmaceutical composition (25 mg/mL) or ranibizumab (22.1 mg/ml) stock to 1mg/g by weight. The 1mg/g solutions were then diluted to 12.8 µg/g. The 12.8 µg/g solution was then diluted to 128 ng/g. The 128 ng/g solution was then diluted by volume in ½ concentration v/v steps to make a 10-point standard curve set 128.0 ng/mL to 0.5 ng/mL along with a with a 0.0 ng/ml background standard. The Manufacturer reports there is no cross reactivity between bevacizumab and any rabbit tissue in this ELISA method.

**4) Animal Tissue Sample Collection**

At the end of the study, rabbits were anesthetized with ketamine/xylazine. Blood was collected via cardiac aspiration. Blood samples were allowed to coagulate, then centrifuged and undiluted serum samples were aliquoted and held at 4°C until ELISA quantification. After blood collection, the animals were euthanized with intra-cardiac saturated potassium chloride.

Eye surfaces, surrounding areas and eye sockets were irrigated several times with substantial amounts of PBS from a pressurized stream from a syringe. The aqueous humor was collected in situ by needle aspiration. Collected samples were placed in the pre-weighed AAF in tissue collection and ELISA diluent formulation and held on wet ice.

After aqueous humor collection, the eyes were enucleated and the whole globes were rinsed with substantial amounts of PBS. The anterior chambers were surgically removed from the globe - exposing the vitreous. The vitreous was removed by (needleless) syringe aspiration. The collected vitreous samples were placed in the pre-weighed AAF in tissue collection and ELISA diluent formulation and held on wet ice.

The eyes were then dissected by making four cuts in the sclera, anterior to posterior, exposing the retinal tissue. Residual vitreous was gently removed from the retina surface and the retina was removed from the globe. The collected retina samples, separated from the choroid, were placed in the pre-weighed AAF in tissue collection and ELISA diluent formulation and held on wet ice.

Each eye was handled independently. Instruments were cleaned and dried after each eye dissection. New surgical fields, gloves, syringes, scalpel blades and all other materials were changed after each eye dissection and tissue collection procedure.

Sample weights and resulting ELISA dilution ratios were then determined. All tissue samples and blood serum were held on wet ice and then refrigerated at 4°C until ELISA quantification. All samples were assayed by ELISA within 48 hours of collection.

**5) Bevacizumab ELISA**

Bevacizumab ELISA assays were performed as per manufacturer’s instructions. The ELISA kit analytical dilution standard material was substituted with the AAF Tissue collection media, and the ELISA calibration standard diluent was substituted with AAF formulation. The analytical calibrator standard was made with the same lot of bevacizumab and diluted with the tissue collection and ELISA diluent AAF formulation. Standard curve samples and tissues samples were plated in duplicate. The assay was run and the optical densities at 450 nm were read on a Biotek (Winooski, VT USA) Synergy H1 plate reader. The optical densities of the two samples were averaged. Standard dilution interpolation curves were drawn from the standard dilution assay set optical densities yielding bevacizumab quantification from the optical density values. Assay bevacizumab sample values were acquired and multiplied by the dilution factor determined by the collection diluent weight and tissue weights. The ELISA method dynamic range for bevacizumab was 0.0 to 200.0 ng/mL. The results are presented as bevacizumab ng/g for tissues and ng/mL for serum.

**6) Ranibizumab Biosimilar ELISA**

Ranibizumab ELISA assays were performed as per manufacturer’s instructions. The ELISA kit analytical dilution standard material was substituted with the AAF Tissue collection media, and the ELISA calibration standard diluent was substituted with AAF formulation. The analytical calibrator standard was made with the same lot of ranibizumab and diluted with the tissue collection and ELISA diluent AAF formulation. Standard curve samples and tissues samples were plated in duplicate. The assay was run and the optical densities at 450 nm were read on a Biotek (Winooski, VT USA) Synergy H1 plate reader. The optical densities of the two samples were averaged. Standard dilution interpolation curves were drawn from the standard dilution assay set optical densities yielding ranibizumab quantification from the optical density values. Assay ranibizumab sample values were acquired and multiplied by the dilution factor determined by the collection diluent weight and tissue weights. The ELISA method dynamic range for ranibizumab was 0.0 to 100.0 ng/mL. The results are presented as ranibizumab ng/g for tissues and ng/mL for serum.
